# A Biomechanical Evaluation of a Novel Airbag Bicycle Helmet Concept for Traumatic Brain Injury Mitigation

**DOI:** 10.3390/bioengineering8110173

**Published:** 2021-11-03

**Authors:** Kwong Ming Tse, Daniel Holder

**Affiliations:** Department of Mechanical and Product Design Engineering, School of Engineering, Swinburne University of Technology, Melbourne, VIC 3122, Australia; 100597544@student.swin.edu.au

**Keywords:** biomechanics, head injury, traumatic brain injury (TBI), expandable bicycle helmet, inflatable bicycle helmet, airbag, helmet design, impact simulations, finite element (FE), protective equipment (PPE)

## Abstract

In this study, a novel expandable bicycle helmet, which integrates an airbag system into the conventional helmet design, was proposed to explore the potential synergetic effect of an expandable airbag and a standard commuter-type EPS helmet. The traumatic brain injury mitigation performance of the proposed expandable helmet was evaluated against that of a typical traditional bicycle helmet. A series of dynamic impact simulations on both a helmeted headform and a representative human head with different configurations were carried out in accordance with the widely recognised international bicycle helmet test standards. The impact simulations were initially performed on a ballast headform for validation and benchmarking purposes, while the subsequent ones on a biofidelic human head model were used for assessing any potential intracranial injury. It was found that the proposed expandable helmet performed admirably better when compared to a conventional helmet design—showing improvements in impact energy attenuation, as well as kinematic and biometric injury risk reduction. More importantly, this expandable helmet concept, integrating the airbag system in the conventional design, offers adequate protection to the cyclist in the unlikely case of airbag deployment failure.

## 1. Introduction

With increasing public awareness for health reasons as well as government promotion, bicycling has become a popular nonmotorised form of transport globally. Nevertheless, road traffic deaths among vulnerable cyclists are intolerably high [1]. It was revealed that approximately 40 thousand bicyclists die in road traffic accidents each year around the world [2], with more than half as a direct result of head injury. Therefore, preventing cyclist head injuries is becoming increasingly urgent for countries such as the Netherlands, Belgium, Denmark, and Australia, where cycling is a common means of commuting.

The bicycle helmet is the only piece of protective equipment for cyclists to provide themselves against traumatic brain injury (TBI) and other head injuries, including facial injuries [3]. The importance of bicycle helmets and their effectiveness in head injury mitigation has been documented in many case studies and research papers [3,4,5,6,7,8,9,10,11,12]. Most of the current bicycle helmets, which are ultimately designed to prevent blunt trauma, e.g., skull fracture, are composed of a soft foam comfort liner and a polymeric foam absorption liner, encased in a thin plastic outer shell with a chin strap for retention in impact. Polymeric foams are the preferred material in absorption liners due to their strong localisation effects in compression and volumetric hardening characteristics. However, in severe cases, the energy-absorbing capability of the conventional liners is insufficient to negate serious brain injury or death [13]. Bicycle helmet absorption liner material is typically expanded polystyrene (EPS), with density ranging from 60–101 kg/m^3^ [14,15]. The efficacy of energy absorption in conventional EPS bicycle helmets has been extensively researched, with evidence suggesting noticeable reduction in head injuries [15,16]. A case study by Bambach et al. [12] indicated that the implementation of an EPS helmet in an impact can produce a reduction in risk of moderate head injury by approximately 49%. Nevertheless, evidence showed that the stiffness of helmet absorption liners in traditional helmet designs is too high in keeping the head deceleration values within the safety thresholds [17,18,19]. Moreover, impact absorption technologies in bicycle helmets remain limited by the liner material properties and overall liner thickness, due to practical and aesthetic concerns. Unlike the safety features, such as passenger airbags and seat belts, implemented in automotive vehicles, there has still been limited improvement in cyclist safety when it comes to crash survivability.

As demonstrated by Meaney and Smith [20] and Willinger et al. [21], there is still inadequate protection by traditional helmets. Therefore, it is necessary to call for in-depth scientific research to escalate the level of bicycle helmet safety [22]. Recently, there is a purportedly increasing number of scientific works on bicycle helmet design. Some researchers are supporting the replacement of the conventional thermoplastic shell with composite materials [23,24], while others have come up with design concepts inspired by natural materials and energy-absorbing structures [22,25,26]. For instance, helmet liners have been suggested to be made up of bioinspired internal structures, mimicking those of pomelo peels, nautilus shells, and woodpeckers’ skulls [22], whilst Kiran Totla et al. [26] proposed a helmet shell inspired by coconut shells. In addition, a Swedish head safety technology company—MIPS (Täby, Sweden), developed and patented a multidirectional impact protection system (MIPS^®^) which utilised multiple slip-plane technology to reduce rotational forces transmitted to the head [27]. Meanwhile, another newly commercialised bicycle helmet, the Trek’s WaveCel^®^ (Waterloo, WI, US) helmet, uses a collapsible helmet concept for rotational acceleration-induced injury mitigation [28], despite recent controversy over its safety claims.

With the availability of high-rate micro-electrical–mechanical system sensors and high energy density batteries, expandable helmet technology has the potential to further reduce cyclist head injury risk; expandable helmets have the ability to sense an impending collision and expand to protect the head. Recently, a wearable expandable head protection device in Sweden was presented to the world in the form of an airbag “helmetless” helmet, known as the Hövding helmet. This device is intended to be worn as a collar that inflates to protect the head when an impact is detected and deemed to have occurred. The Hövding design has shown, through independent studies, improvements in linear and rotational energy attenuation and overall injury risk in common impact scenarios when compared to a range of EPS helmet types [29,30]. Issues arise with this design surrounding no protection in a failure to deploy, the potential to bottom out under high blunt force, and the overall design failing to meet the majority of international bicycle helmet standards, such as EN 1078. Since this design was released in 2017, there has been no research into alternative airbag bicycle helmet designs and the consequential effect on TBI. Expandable protection devices have shown a global capacity to dissipate inertial forces in impact scenarios. While the concept of expandable protection alone is not new, with research in areas such as wearable fall protection devices and automotive airbags, the potential for this technology in cyclist protection remains relatively unexplored. Furthermore, there is a paucity of research investigating how expandable technologies can assist in injury mitigation for cyclists. Kurt et al. [29] explored the potential for optimisation of inflation pressure of the Hövding 2.0 expandable protection device in impact scenarios and found that the “helmetless” device has nearly seven-fold reduction in head injury criterion (HIC) value when compared to that of traditional EPS helmet designs when inflated to 72 kPa.

In this study, the authors explore the synergetic features of an expandable airbag and a standard commuter-type EPS helmet in their expandable helmet design. Since most instances of serious harm and death in cyclists are attributed to injuries pertaining to the head and neck, the authors conceptualised a novel expandable helmet, comprising an exemplary conventional EPS helmet, namely, Pro-Tec’s The Classic EPS (Pro-Tec, Melbourne, Victoria, Australia)—and an airbag system that will be deployed prior to impact, will protect the vital regions vulnerable to fatal head and neck injuries. This expandable helmet enhances the energy-absorbing capability by increasing contact area and impact duration, while maintaining a form factor compliant with international standards. To the authors’ best knowledge, this conceptual idea of incorporating an airbag system in the traditional bicycle helmet is completely new and does not have precedent elsewhere. The effectiveness of this novel “airbag helmet” concept against injury risk is examined against that of the exemplary conventional EPS helmet, using a computational approach.

## 2. Materials and Methods

In the present study, a series of four dynamic impact simulations were carried out on both the helmeted headform and head models with different configurations, using Ansys Workbench LS-DYNA v.192 (ANSYS, Inc., Canonsburg, PA, USA) (Table 1). The impacts were performed initially on a ballast headform for validation and benchmarking, while the subsequent impacts were conducted on a biofidelic finite element (FE) model of human head and were used for assessing intracranial injuries.

### 2.1. The Finite Element Model

**Figure 1:**
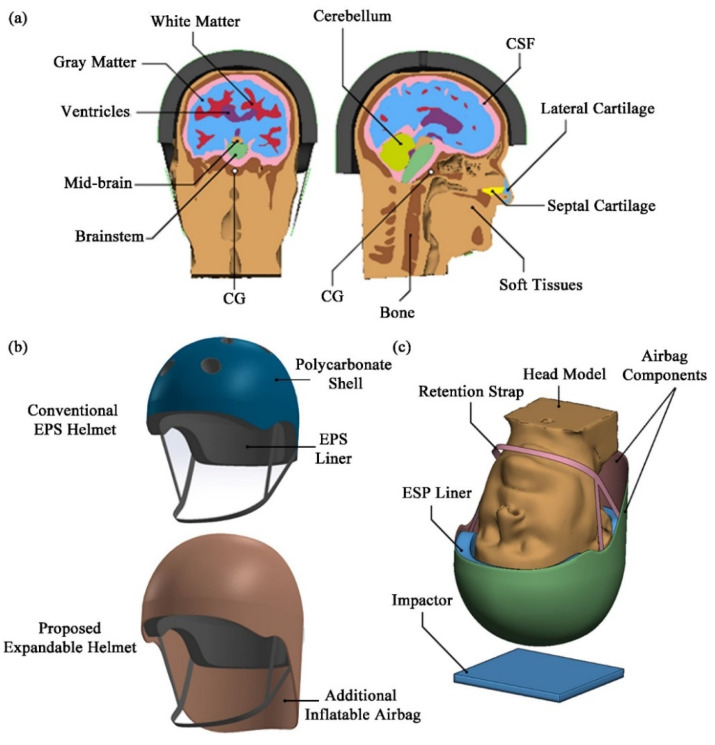
(**a**) Various components of the head model; (**b**) the exemplary conventional EPS helmet and expandable helmet; (**c**) various components of the expandable helmet model in the impact scenario in accordance with EN 1078 standard (1997).

The FE model of human head and brain used in the current study comprises the skeletal skull, the cerebrospinal fluid (CSF), the white and grey matters of cerebrum, cerebellum, the ventricular system, the midbrain, and the brain stem, as well as the overlying soft tissue (Figure 1a). It should be noted that the FE head model had previously been validated against the intracranial pressure (ICP) and relative displacement data of three cadaveric experiments [31]. More details on the development and validation of the head model, as well as its material properties, can be found in Tse et al. [31].

The expandable helmet was designed around a standard commuter-type bicycle helmet which includes an EPS liner encased in a thin polycarbonate (PC) shell and a polyethylene (PET) retention strap (Figure 1b). In addition to the traditional EPS helmet, the proposed airbag helmet has a novel and unique expandable feature that inflates upon activation, encompassing the entire helmet and posterior neck in a top-down manner (Figure 1c). Nonlinear material properties, such as the volumetric stress-strain data of EPS from [32], were used to define the properties of the EPS liner in our study using empirical data. Material properties of other bicycle helmet components were obtained from the literature, as outlined in Table 2, whereas the expandable airbag was assumed to be of Nylon 6-6 (Polyamide 66 or PA66), as is typical in the automotive industry [33]. The dimensions of the inflatable or “airbag” component before activation and after being fully inflated are shown in Appendix A. This inflatable component, along with the exterior helmet shell and retention strap, were modelled with four-noded quadrilateral shell elements, while the helmet liner was represented by eight-noded hexahedral elements.

**Table 2:** Material properties of various components in the airbag–helmet–headform model.

Components	Material Properties	Thickness [mm]	LS-DYNA Material Model	Ref.
Retention Strap (PET)	ρ = 1400 kg/m^3^ E = 1000 MPa ν = 0.44	1.5	MAT_ELASTIC_001	Milne et al. [32]
Liner (EPS)	ρ = 61.6 kg/m^3^ E = 28 MPa ν = 0.01	20	MAT_ CRUSHABLE_FOAM_063	Milne et al. [32]
Helmet Shell (PC)	ρ = 1055 kg/m^3^ E = 1500 MPa ν = 0.42	0.4	MAT_ELASTIC_001	Deck and Willinger [34]
Airbag Component (Nylon 6-6 or Polyamide 66)	ρ = 1000 kg/m^3^ E =100 MPa ν = 0.40	0.35 (fabric thickness)	MAT_FABRIC_034	Avula et al. [35]
Anvil (Steel)	ρ = 7830 kg/m^3^ E =207,000 MPa ν = 0.30	24	MAT_RIGID_ 020	Sandberg et al. [36]
Headform (MgK1A)	ρ = 1740 kg/m^3^ E =44,800 MPa ν = 0.32	N/A	MAT_RIGID_ 020	SA/SNZ [37]

### 2.2. Impact Simulation Environment, Interaction, Initial and Boundary Conditions

The dynamic impact simulations were designed to replicate the impact testing with a flat anvil, as described in European standard, EN 1078. For the purpose of model validation, the impact location directly on top of the helmet was selected to best replicate conditions shown by Sandberg et al. [36], in which an identical model was used. The flat steel anvil was modelled as a rigid plate, which was constrained in all six DOFs, whereas the initial velocity of the helmet–headform/head model was set to 5.42 m⋅s^−1^ to simulate the terminal velocity of a 1.5 m drop with a 4.73 kg head and 0.2 kg helmet, as outlined in EN 1078 standard.

To ensure appropriate interactions between the intracranial components, CSF and the skull, a global AUTOMATIC_SINGLE_SURFACE_TIEBREAK contact definition was set, encompassing all the components of the head model. This contact formulation allows tangential motion and sliding with friction, following similar suggestions made by Kleiven and Hardy [38]. In each case, AUTOMATIC_SURFACE_TO_SURFACE contacts were defined between the helmet shell/airbag surface and the impactor, the EPS liner and the impactor, and the headform (or head) and the helmet. TIED_SURFACE_TO_SURFACE contacts were applied between the helmet shell and the outer EPS surface as well as between the retention strap and the outer shell, to ensure no relative motion. The kinetic friction coefficient was assumed to be of a typical value of 0.2 globally, while the kinetic friction coefficient between the impactor and the helmet shell/airbag was set to be 0.1, as recommended in [35].

The airbag was defined using the AIRBAG_HYBRID material model in LS-DYNA, in which the fluid in the airbag was defined as air with an atmospheric density, pressure, and molar gas constant of 1.28 kg⋅m^−3^, 101.325 kPa, and 8.314 J⋅K^−1^⋅mol^−1^, respectively, using typical values for air at 0 °C [39]. The initial state of the airbag was defined with initial molecular weight of 28.8 g⋅mol^−1^ and inflation molecular weight of 22.5 g⋅mol^−1^ [39]. Mass input and output flow rate was defined in such a way that it resulted in an inflation airbag pressure of approximately 72 kPa upon impact, which is identical to the optimised airbag pressure reported by Kurt et al. [29].

### 2.3. Injury Assessment and Evaluation Method

Head injury is typically classified according to the injury site, whether it is extracranial (skull) or intracranial (brain tissues). Previous studies have shown that intracranial injuries, such as diffuse axonal injury (DAI), are often caused by tissue shearing induced by rotational kinematics, while linear acceleration induces compressive skull fractures [31,32]. One commonly used criterion of linearly induced injury is stated in the European EN 1078 standard, that forbids peak acceleration exceeding 250 G and the duration of the acceleration above 150 G being more than 6 ms, while another common evaluation metric of linearly induced head injury is the HIC, shown in Equation (1). The HIC value is used indicate to the probability of injury at levels of the Abbreviated Injury Scale (AIS), i.e., a six-tier injury classification scale, with AIS level 1 characterising minor injury and AIS level 6 indicating maximal injury (untreatable).

(1) $$\mathrm{HIC}={\{\left(t_{2}-t_{1}\right)\cdot {\left[\frac{1}{t_{2}-t_{1}}\int_{t_{1}}^{t_{2}}a\left(t\right)dt\right]}^{2.5}\}}_{\mathrm{MAX}}$$

Besides the HIC, there are other metrics for quantifying TBI in the literature; the ICP criterion is amongst the most common brain injury criteria, with pressures ranging within 173–235 kPa indicating potential moderate level brain injury [11]. Along with the ICP criterion, maximum principal strain (MPS) within the brain tissue is also commonly used to indicate potential injury, with MPS values of 0.1–0.2 typically considered the threshold for potential irreversible tissue damage [35]. Further, a method of predicting the likelihood of a particular AIS level of brain injury using the MPS value has been proposed by Takhounts et al. [40], with AIS level 3 typifying serious brain injury, as shown in Equation (2).

(2) $$P\left(\mathrm{AIS}\;3\right)=1\;-e^{{\left(\frac{\mathrm{MPS}}{0.828}\right)}^{2.84}}$$

In the present study, the impact simulation of the helmeted ballast headform was used for validation against an identical experimental impact test, while in subsequent impacts, the linear acceleration at the centre of gravity (CG) of the biofidelic human head model was extracted to quantify HIC risk factor. In addition to the head kinematic responses, intracranial injury metrics, such as ICP and MPS, were taken from the representative head model to investigate the likelihood of traumatic brain injury, as well as to identify the potential injured sites within the intracranial tissues.

## 3. Results

### 3.1. Indirect Validation and Benchmarking against Experimental Impacts on the Ballast Headform

This study adopted an indirect validation approach in which the headform kinematic of the helmeted ballast headform impact was first validated before assessing the performance of the proposed expandable bicycle helmet, in terms of the unobtainable intracranial biomechanical responses, using a previously validated biofidelic head model [31]. It should be highlighted that this indirect validation is unavoidable, as the required cadaveric experimental tests on human heads are cost-prohibitive, difficult to perform, and involve ethical issues.

**Figure 2:**
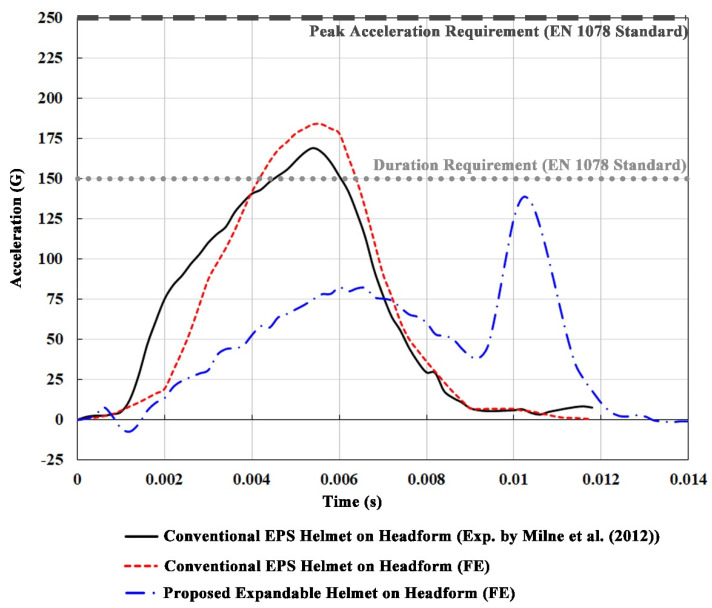
Experimental and simulated accelerations at the CG of the headform equipped with the EPS control helmet and the novel airbag helmet.

The predicted time history of the linear acceleration at the CG of the ballast headform model equipped with the conventional EPS helmet was used to benchmark and compare with the experimentally obtained data from the helmeted headform impact by Milne et al. [32], using the identical impact scenario in accordance with EN 1078 standard (1997). It can be seen in Figure 2 that both the EPS-only helmet and the proposed expandable helmet passes the EN 1078 standard, with no peak linear acceleration exceeding 250 G and the duration of the acceleration above 150 G being less than 6 ms. Moreover, the predicted linear acceleration at the CG of the headform agreed very well with the experimental data, with coefficient of determination (R^2^) of 0.925 and a difference in peak acceleration value of 9.6% (simulation: 185 G; experiment: 170 G). In addition, the simulated peak acceleration was comparable (16.7% higher) than the experimental value obtained by Cripton et al. [9], with a similar drop height and a contemporary CCM V15 Backtrail bicycle helmet.

Furthermore, the same ballast headform was equipped with our expandable “airbag” helmet and was impacted in the same conditions to provide a performance benchmark for the concept against the experimental data. When compared to the EPS-helmeted-headform kinematics, the expandable helmet substantially reduced the head acceleration by 57.8% in its first acceleration peak, despite giving rise to a second peak of 140 G, at which point the airbag is completely deflated and the helmet finally contacts the anvil. The corresponding decline in HIC_36_ was approximately 70% (from 1186 to 355), whereas the peak contact force between the headform and the helmet liner dropped from 8.45 kN to 6.15 kN.

### 3.2. Injury Assessment Using a Validated Biofidelic Human Head and Brain Model

This indirect validation of the head kinematic response from the numerical simulations of the helmet–headform impact allows us to further examine the effectiveness of our expandable bicycle helmet, in terms of other biomechanical injury metrics, which cannot be obtained from the helmet–headform simulation. With the validated biofidelic human head and brain model, intracranial biomechanical parameters, in particular, the intracranial pressure (ICP) and maximum principal strain (MPS), were determined and compared with various injury criteria data reported in the literature.

**Figure 3:**
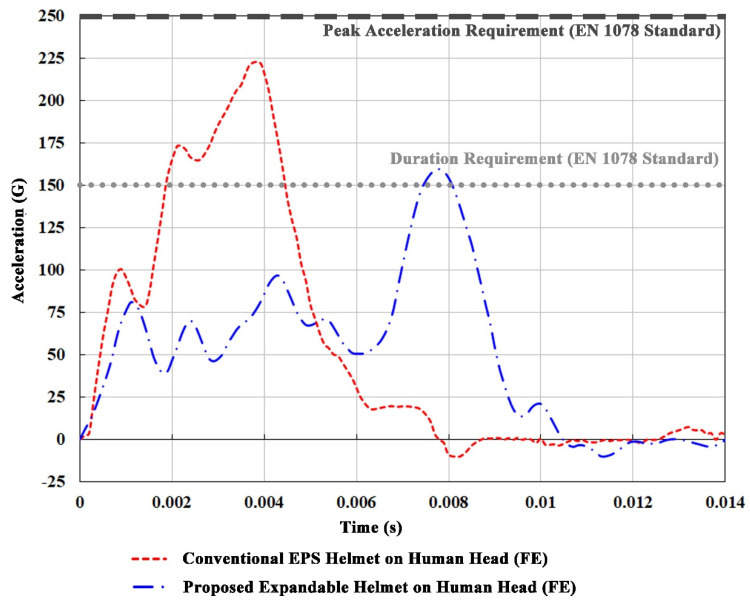
The acceleration histories at the CG of the head model for the EPS helmet and the proposed expandable helmet.

Figure 3 shows the linear accelerations predicted at the CG of the biofidelic head model when it is equipped with the expandable helmet in comparison with the EPS-only helmet. It should be noted that, consistent with previous ballast headform results, both the conventional helmet and the proposed expandable helmet fulfill the EN 1078 standard, and there is a substantial reduction in linear acceleration of approximately 26.5% (from 219 G to 161 G) at the CG of the head model. The duration of the impulsive acceleration is significantly longer, hence reducing the HIC_36_ value from 1170 to 536. This decrease in HIC_36_ resulted in a reduction of the probability of AIS level 3 (moderate) injury from 64% to 17%, based on injury curves developed by Mertz et al. [41].

**Figure 4:**
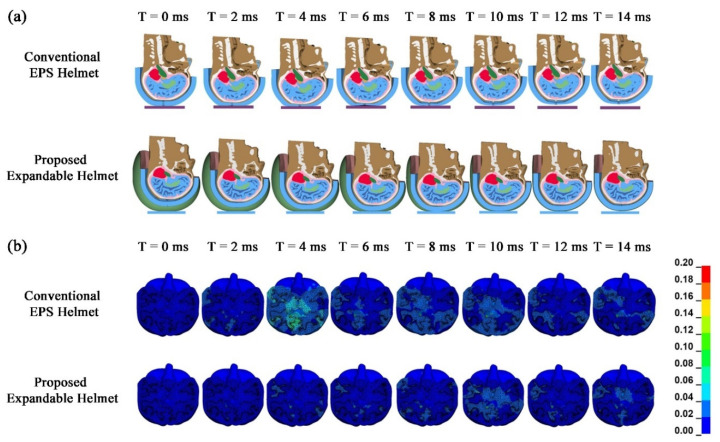
Comparison of the EPS-only helmet and the expandable-airbag EPS helmet. (**a**) Transient response and (**b**) maximum principal strain (MPS).

**Figure 5:**
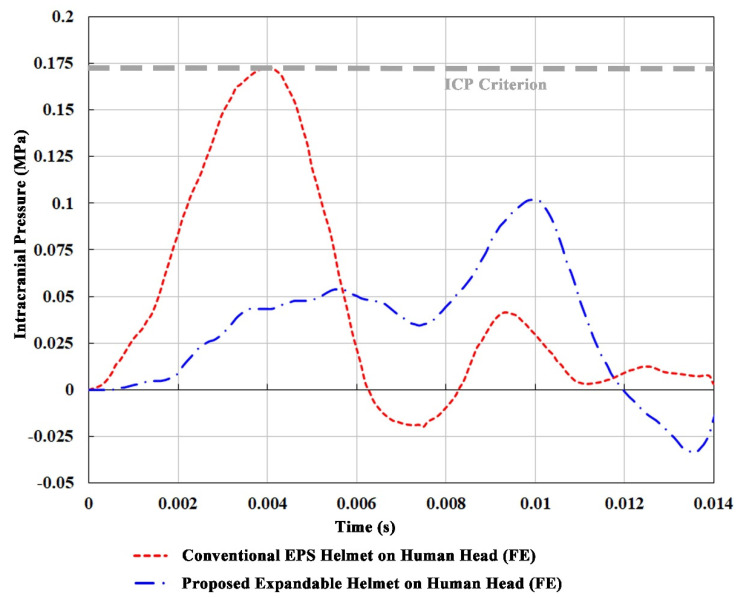
The intracranial pressure (ICP) histories at the mid-brain region of the head model for the EPS helmet and the proposed expandable helmet.

Average ICP taken across tissues in the cerebral cortex region shows a 41% drop in peak value from 0.173 MPa to 0.10 MPa, as shown in Figure 4 and Figure 5. This indicates the potential to reduce the risk of moderate brain injury to minor or no brain injury. The case of the EPS helmet showed that the average MPS was 0.08–0.10 with a centroid sectional peak of 0.40 located in the interface of the white matter and grey matter in the central cerebrum, outlined in Figure 4. These MPS values are comparable to those predicted in Fahlstedt et al. [42]’s FE study using three accident reconstruction cases. On the contrary, when the head was equipped with the proposed expandable helmet, the average MPS was found to be in the range of 0.02‒0.04, with the peak MPS values of around 0.26 in the same central cerebrum region in the brain. Nevertheless, both cases show the likelihood of reversable and irreversible injury according to Tse et al. [43] and Tse [44]; using the maximum MPS value in each case shows that the probability of AIS 3 brain injury decreased from 11.9% to 3.7% when the proposed expandable helmet design was implemented.

## 4. Discussion

The additional inflatable “airbag” feature of the expandable helmet proposed in this study provides an additional crumple zone, as the inflated airbag is designed to deform upon impact, in order to prevent jarring to the cyclist’s head and neck. To explore the synergetic effect of an expandable airbag and a standard commuter-type EPS helmet in the novel helmet design, a series of dynamic impact simulations were performed on a human head model equipped with the proposed novel expandable helmet, comprising the conventional EPS helmet and an airbag, before the helmet was evaluated for its efficacy in protecting the bicyclist’s head against TBI. The validity of the numerical modelling of these dynamic impact events was confirmed by their comparison with experimental impact in a controlled laboratory setting, compliant with European EN 1078 standard (1997).

Our proposed expandable helmet was found to have better energy-absorbing capability, as compared to the conventional EPS helmet, with a lower maximum force exerted on the head, and yet not exceeding the helmet liner’s deformation limit where its bottoming-out occurs. It was concluded that the additional inflatable “airbag” feature encompassing the EPS helmet is more effective in attenuating impact energy by providing a primary energy-absorbing surface between the impacting object and the helmeted head, as compared to the conventional EPS bicycle helmet, and thus is most likely to reduce concussion. The expandable helmet minimised the peak acceleration experienced by the head by extending the time duration of the impulsive impact and further delaying the occurrence of the peak acceleration. This resulted in a substantial reduction in HIC_36_, from 1170 when equipped with the EPS-only helmet to 536 for the expandable helmet. This HIC_36_ reduction corresponds with a decrease in the probability of AIS level 3 head injuries (from 64% to 17%), based on injury curves developed by Mertz et al. [41]. It is noteworthy to mention that there is a secondary spike in acceleration pulse at around T = 8 ms, when the head was equipped with the airbag helmet (Figure 3). This is attributed to the exterior helmet shell coming into contact with the impactor when the airbag bottoms out. It should be highlighted that this bottom-out of the airbag was prearranged and purposeful, as this is the fundamental trade-off between the maximum force exerted on the head and the helmet liner’s deformation limit, in helmet design.

In regard to the intracranial injuries, the most common biomechanical parameters for evaluating TBI are induced by both the translational and rotational acceleration, according to the skull-deformation–angular acceleration theory [45]. The hypotheses that pure translational acceleration creates ICP gradients which may result in regional variation in cerebral blood flow for long duration impact [46], whereas pure rotational acceleration causes rotation of the skull relative to the brain, which is likely to tear parasagittal bridging veins [47], were widely accepted within the scientific community, and therefore both the intracranial biomechanical parameters were included for injury assessment. When compared to the traditional EPS helmet, the expandable helmet recorded a lower ICP and MPS, indicating the superiority of the airbag helmet in intracranial injury mitigation.

Due to the parametric nature of this study, the results presented provide qualitative indication of the performance of the novel expandable helmet as compared to the conventional helmet. The purpose of this study is not to evaluate the performance of the current expandable helmet design with the Hövding “helmetless” airbag-only concept. Contrastingly, the authors of this study aim to explore and investigate the synergetic effect of an expandable airbag and a standard commuter-type EPS helmet in injury mitigation, with the goal of minimising the risk of injuries, while there still exists a possibility of failed airbag deployment due to any possible electromechanical or algorithmic fault of the trigger unit. More importantly, since most instances of serious harm and death in cyclists are attributed to injuries pertaining to the head and neck, the authors conceptualised that the proposed expandable helmet, which integrates an airbag system into the conventional design, will still offer adequate protection to cyclists, even if the airbag fails to deploy, and protect the vital regions vulnerable to fatal head and neck injuries.

## 5. Conclusions

In summary, the current EPS helmet design provides adequate protection; however, there is potential for further technological enhancement in reducing the risk of a concussion or other serious brain injury. The novel expandable helmet design presented performed admirably when compared to a conventional EPS helmet design—showing improvements in overall attenuation capacity in impact energy and kinematic and biometric injury risk factors. This may be deemed as a more adequate and conservative approach to airbag helmet technologies, as compared to the airbag-only design, especially if the airbag fails to deploy due to any potential electromechanical triggering issue. Conclusively, this study intends to answer research questions related to the level of injury risk reduction seen in the revolutionary airbag design when compared to a conventional EPS helmet, with motivations to promote further research on helmet design and optimisation.

## Figures and Tables

**Table 1:** Dynamic simulations with various impact scenarios and configurations.

Case No.	Impact Scenario & Configuration	
1	Conventional EPS Bicycle Helmet on Ballast Headform	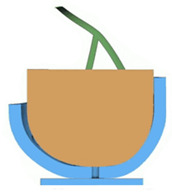
2	Proposed Expandable Bicycle Helmet on Ballast Headform	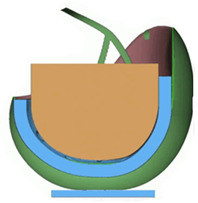
3	Conventional EPS Bicycle Helmet on Human Head	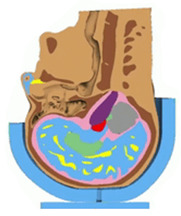
4	Proposed Expandable Bicycle Helmet on Human Head	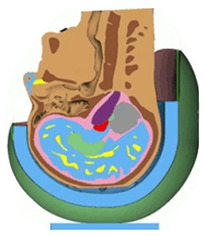

**Figure A1:**
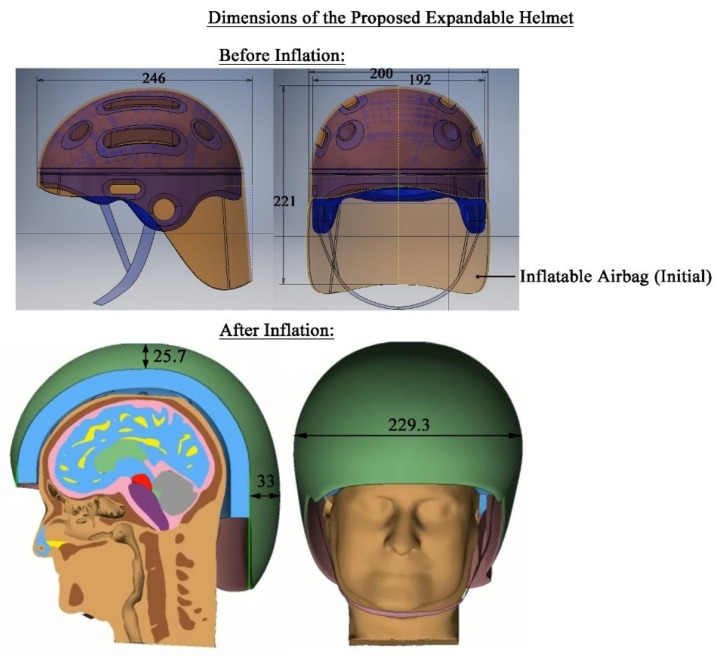
Dimensions of the proposed expandable helmet (before and after inflation).
